# Supplementary figures and images for: Assessing the value and knowledge gains from an online tick identification and tick-borne disease management course for the Southeastern United States
Source: BMC Public Health. 2024 Jul 5;24:1793. doi: 10.1186/s12889-024-19307-x (PMC11225117; doi:10.1186/s12889-024-19307-x)

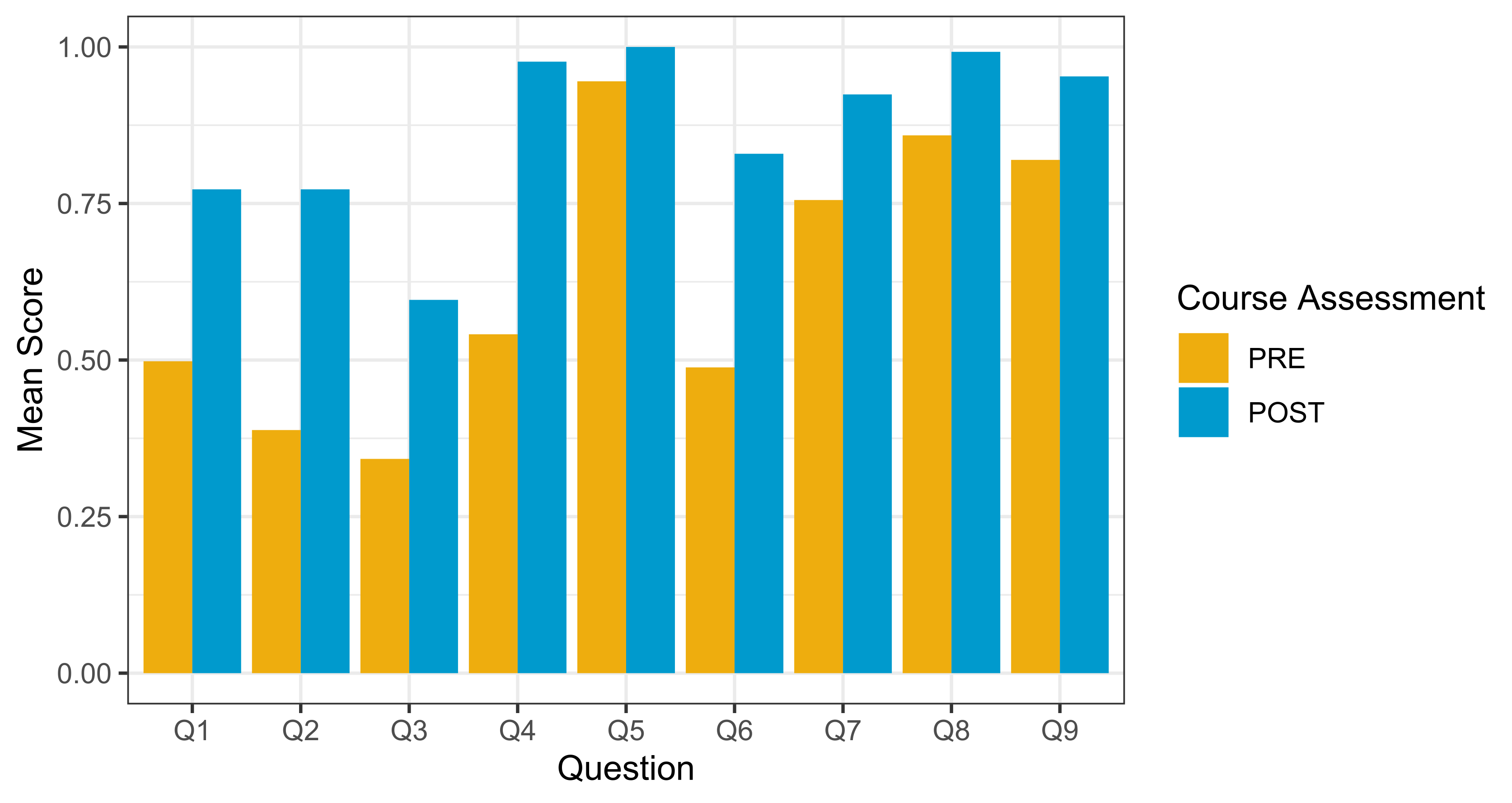

Supplement: Supplementary file 1 — Supplementary Material 1 [file 12889_2024_19307_MOESM1_ESM.tif]
