# Supplementary material for: Assessing the value and knowledge gains from an online tick identification and tick-borne disease management course for the Southeastern United States
Source: BMC Public Health. 2024 Jul 5;24:1793. doi: 10.1186/s12889-024-19307-x (PMC11225117; doi:10.1186/s12889-024-19307-x)

**S1 Table.** Questions and multiple-choice answer choices included in the post-course student assessment. In addition to these questions, the full assessment also included several sections for students to provide feedback via open text responses.

| **Q: Please select the response below that best describes your role within your pest management services organization** |
| --- |
| *Certified operator*  *Supervisor of technicians*  *Public health specialist (e.g., environmental scientist, state entomologist, entomologist)*  *Military entomologist*  *Other (specified in open text)* |
| **Q: Please describe your current engagement in tick surveillance and control activities (select all that apply)** |
| *I supervise others who are directly involved in tick surveillance and/or control activities*  *I am directly involved in tick surveillance and/or control activities*  *My working unit is responsible for tick surveillance and/or control, but I am not directly involved in these activities*  *My work is on human disease surveillance for tick-borne diseases*  *My working unit is not directly involved in or connected to tick surveillance and/or control activities*  *Other (specified in open text)* |
| **Q: Rate your knowledge of (or your skill in) the following (Tick Biology, Tick Identification, Tick Surveillance, Tick Control, Tick-borne Diseases, Tick Safety, Ticks and Public Health) BEFORE the course:** |
| *Not at all knowledgeable*  *Slightly knowledgeable*  *Moderately knowledgeable*  *Very knowledgeable*  *Extremely knowledgeable* |
| **Q: Rate your knowledge of (or your skill in) the following (Tick Biology, Tick Identification, Tick Surveillance, Tick Control, Tick-borne Diseases, Tick Safety, Ticks and Public Health) AFTER the course:** |
| *Not at all knowledgeable*  *Slightly knowledgeable*  *Moderately knowledgeable*  *Very knowledgeable*  *Extremely knowledgeable* |
| **Q: How relevant is this course to your current work?** |
| *Not at all relevant*  *Slightly relevant*  *Moderately relevant*  *Very relevant*  *Extremely relevant* |
| **Q: What is your opinion of the balance of lecture and interactivity in this course?** |
| *Too much lecture and not enough interactive learning*  *Right amount of both lecture and interactive training*  *Too much interactive and not enough lecture* |
| **Q: Will you use what you learned in this course in your work?** |
| *Definitely will*  *Probably will*  *Possibly*  *Probably will not*  *Definitely will not*  *Not applicable, I did not learn anything new in this course* |
| **Q: What factors will keep you from using the content of this course in your work? Select all that apply.** |
| *I will not have the resources I need*  *I will not be provided opportunities to use what I learned*  *I will not have the time to use what I learned*  *My supervisor will not support me in using what I learned*  *My colleagues will not support me in using what I learned*  *The course content is not relevant to my current work*  *Other (please specify in next question)* |

Figure S1. Comparison of average paired scores on questions from participants (n=255) who completed both the pre-course (orange) and post-course (blue) OTTC assessments.


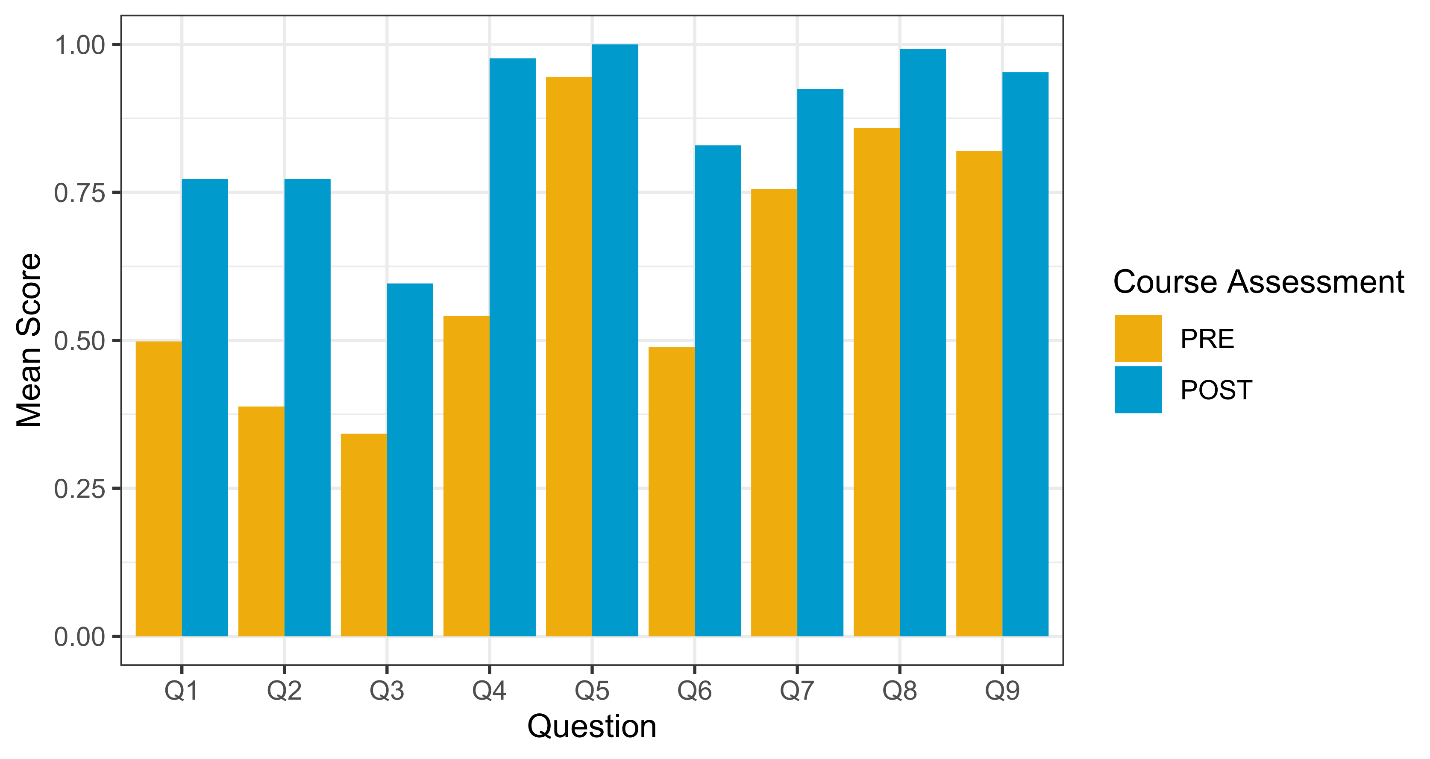

Supplement: Supplementary file 2 — Supplementary Material 2 [file 12889_2024_19307_MOESM2_ESM.docx]
