# Supplementary material for: Assessing the value and knowledge gains from an online tick identification and tick-borne disease management course for the Southeastern United States
Source: BMC Public Health. 2024 Jul 5;24:1793. doi: 10.1186/s12889-024-19307-x (PMC11225117; doi:10.1186/s12889-024-19307-x)
Supplement: Supplementary file 3 — Supplementary Material 3 [file 12889_2024_19307_MOESM3_ESM.docx]

S2 Table. OTTC post-course student survey questions.

| **Q1: Please select the response below that best describes your role within your pest management services organization.** |
| --- |
| o Certified operator  o Supervisor of technicians  o Public health specialist (e.g., environmental scientist, state entomologist, entomologist)  o Military entomologist  o Other (please specify in next question) |
| **Q2: If you selected "other" in the previous question about your role within a pest management services organization, please specify your role below.**    **If you did not select "other" in the previous question, please enter "N/A" below.** |
| Open text response |
| **Q3: Please describe your current engagement in tick surveillance and control activities (select all that apply)** |
| o I supervise others who are directly involved in tick surveillance and/or control activities  o I am directly involved in tick surveillance and/or control activities  o My working unit is responsible for tick surveillance and/or control, but I am not directly involved in these activities  o My work is on human disease surveillance for tick-borne diseases  o My working unit is not directly involved in or connected to tick surveillance and/or control activities  o Other (please specify in next question) |
| **Q4: If you selected "other" in the previous question describing your current engagement in tick surveillance and control activities, please specify below.**    **If you did not select "other" in the previous question, please enter "N/A" below.** |
| Open text response |
| **Q5: Rate your knowledge of (or your skill in) the following before the course:**    **Tick Biology, Tick Identification, Tick Surveillance, Tick Control, Tick-borne Diseases, Tick Safety, Ticks and Public Health** |
| o Not at all knowledgeable  o Slightly knowledgeable  o Moderately knowledgeable  o Very knowledgeable  o Extremely knowledgeable |
| **Q6: Rate your knowledge of (or your skill in) the following after the course:**    **Tick Biology, Tick Identification, Tick Surveillance, Tick Control, Tick-borne Diseases, Tick Safety, Ticks and Public Health** |
| o Not at all knowledgeable  o Slightly knowledgeable  o Moderately knowledgeable  o Very knowledgeable  o Extremely knowledgeable |
| **Q7: How relevant is this course to your current work? (selected from drop-down menu)** |
| o Not at all relevant  o Slightly relevant  o Moderately relevant  o Very relevant  o Extremely relevant |
| **Q8: What is your opinion of the balance of lecture and interactivity in this course? (selected from drop-down menu)** |
| o Too much lecture and not enough interactive learning  o Right amount of both lecture and interactive training  o Too much interactive and not enough lecture |
| **Q9: Will you use what you learned in this course in your work? (selected from drop-down menu)** |
| o Definitely will  o Probably will  o Possibly  o Probably will not  o Definitely will not  o Not applicable – I did not learn anything new from this course |
| **Q10: What, if anything, do you plan to use from this course?** |
| Open text response |
| **Q11: What factors will keep you from using the content of this course in your work? Select all that apply.** |
| o I will not have the resources I need  o I will not be provided opportunities to use what I learned  o I will not have the time to use what I learned  o My supervisor will not support me in using what I learned  o My colleagues will not support me in using what I learned  o The course content is not relevant to my current work  o Other (please specify in next question) |
| **Q12: If you selected "other" in the previous question about the factors which will keep you from using the content of this course in your work, please specify below.**    **If you did not select "other" in the previous question, please enter "N/A" below.** |
| Open text response |
| **Q13: How could this course be improved to make it a more effective learning experience?** |
| Open text response |
| **Q14: What part of this course was the most helpful to your learning?** |
| Open text response |
